# Adverse events following Synflorix vaccination reported to the Vaccine Adverse Event Reporting System (VAERS), 2010–2024

**DOI:** 10.1371/journal.pone.0338640

**Published:** 2026-01-12

**Authors:** Lingyun Cui, Nian Tong, Sicong Hou, Chun Yu

**Affiliations:** 1 The First School of Clinical Medicine, Faculty of Medicine, Yangzhou University, Yangzhou, PR China; 2 Key Laboratory of the Jiangsu Higher Education Institutions for Integrated Traditional Chinese and Western Medicine in Senile Diseases Control (Yangzhou University), Yangzhou, PR China; 3 Department of Gastroenterology, Affiliated Hospital of Yangzhou University, Yangzhou University, Yangzhou, Jiangsu, China; 4 Department of Pediatrics, Affiliated Hospital of Yangzhou University, Yangzhou University, Yangzhou, PR China; Maitama District Hospital, NIGERIA

## Abstract

**Background:**

Synflorix is a conjugate vaccine targeting Streptococcus pneumoniae, effectively reducing pneumonia and invasive pneumococcal disease in children under five. To evaluate its safety profile, this study analyzed adverse event (AE) reports associated with Synflorix vaccination submitted to the Vaccine Adverse Event Reporting System (VAERS) from 2010 to 2024.

**Methods:**

We conducted a retrospective analysis of 1,704 VAERS reports from January 1, 2010, to December 30, 2024. Data processing and statistical analysis were performed using R software. Reporting Odds Ratios (ROR) with 95% confidence intervals (CI) were calculated to identify potential safety signals. Symptom types, onset times, and severity differences across age groups were examined. To facilitate comparison with other vaccines in the VAERS database, certain Preferred Terms (PTs) were normalized per 100,000 reports.

**Results:**

The most common adverse reactions included crying, pyrexia, hypotonic-hyporesponsive episodes (HHE), pallor, and diarrhea. Newly detected signals not previously listed in the product insert were HHE, hypotonia, pallor, and bronchiolitis. Severe reactions such as pyrexia, crying, vomiting, and pallor showed delayed onset compared to non-severe cases. Infants and toddlers demonstrated stronger systemic reactions within one day post-vaccination, while children aged 2–10 years mostly developed injection site nodules. Adults predominantly experienced localized reactions within 10 days. Reports for elderly individuals (≥55 years) were limited and primarily described mild symptoms like erythema and pyrexia. When compared to other pneumococcal vaccines (PCV7, PCV13, PCV15, PCV20, PPSV23), Synflorix showed the third-highest frequency of mortality and significantly higher RORs for HHE, hypotonia, pallor, and bronchiolitis. Additionally, in terms of the timing of AEs, Synflorix exhibited a similar trend to these vaccines, with reactions primarily occurring in the short-term post-vaccination period.

**Conclusion:**

Age-specific patterns of adverse events following Synflorix vaccination were observed, with infants and toddlers showing higher intensities of systemic reactions. Compared to other pneumococcal vaccines, Synflorix exhibited a relative higher mortality reporting frequency, along with higher frequencies and RORs for specific PTs, including HHE, hypotonia, pallor, and bronchiolitis. Enhanced post-vaccination monitoring is recommended for younger populations due to their immature immune systems. Age-stratified surveillance can help optimize vaccine safety and management.

## Introduction

According to the World Health Organization’s (WHO) 2019 report, pneumonia remains one of the leading causes of death among children under five worldwide, accounting for approximately 700,000 deaths annually, particularly in developing countries [1]. This significant burden presents a major challenge to global public health systems [2]. Therefore, the prevention and control of Streptococcus pneumoniae, a primary pathogen responsible for pneumonia, are critical. Studies have shown that prophylactic immunization with pneumococcal conjugate vaccines (PCVs) significantly reduces the incidence and mortality of pneumonia caused by vaccine-included serotypes, thereby easing the strain on healthcare resources [3]. Synflorix, a ten-valent pneumococcal conjugate vaccine developed by GlaxoSmithKline (GSK), is indicated for infants and children aged 6 weeks to 5 years. It targets ten common Streptococcus pneumoniae serotypes (1, 4, 5, 6B, 7F, 9V, 14, 18C, 19F, and 23F) [4]. Since its initial licensure in the European Union and other regions in 2009, it has played an important role in global healthcare [5]. Both clinical trials and real-world effectiveness studies have consistently demonstrated that Synflorix provides over 90% protective efficacy against invasive pneumococcal disease [6].

Despite these successes, Synflorix’s safety profile remains a key concern among clinicians and the public, particularly in immunologically immature pediatric populations [7]. Existing evaluations, primarily based on systematic reviews and retrospective cohort studies, report a favorable tolerability profile, with most adverse events (AEs) being transient and mild, such as low-grade fever, redness, and pain at the injection site [8,9]. Nevertheless, these studies are often limited by single-center settings, small sample sizes, and narrow geographic and ethnic representation, which may not fully capture the vaccine’s safety in broader populations [10]. Established in 1990 by the US Centers for Disease Control and Prevention (CDC) and co-managed with the US Food and Drug Administration (FDA), the Vaccine Adverse Event Reporting System (VAERS) aggregates reports of post-vaccination AEs around the world [11]. As a comprehensive passive surveillance system, VAERS provides timely data for the early detection of potential safety signals and has consistently helped public health authorities refine vaccine policies. Herein, we systematically analyze VAERS reports of adverse events following Synflorix vaccination from 2010 to 2024, and compared them with reports for other common pneumococcal vaccines in the VAERS database (PCV7, PCV13, PCV15, PCV20, PPSV23). Our research objective is to characterize the types and temporal patterns of AEs, identify novel signals, such as HHE, hypotonia, pallor, and bronchiolitis, and delineate age-specific onset profiles to inform optimized vaccination strategies and improve safety management across diverse populations.

## Materials and methods

### 1. Data source

The data for this study were obtained from the VAERS online database, including the VAERSDATA, VAERSSYMPTOMS, VAERSVAX files for the years 2010–2024, and Non-Domestic VAERS data. Each report includes patient demographics, vaccination and AE dates, detailed descriptions of the events, past medical history, comorbidities, clinical findings, and diagnostic outcomes [12,13]. AEs are coded using MedDRA Preferred Terms (PTs), with up to five PTs recorded for each VAERS ID [11]. Serious adverse events, as defined in Title 21 of the Code of Federal Regulation (CFR), section 600.80, include outcomes of death, life-threatening conditions, inpatient hospitalization or prolongation of existing hospitalization, persistent or significant disability or incapacity, and congenital anomaly or birth defect; events judged medically important may also be considered serious. The full Synflorix package insert is available at: https://ca.gsk.com/media/6260/synflorix.pdf.

### 2. Patient cohort

**Fig 1:**
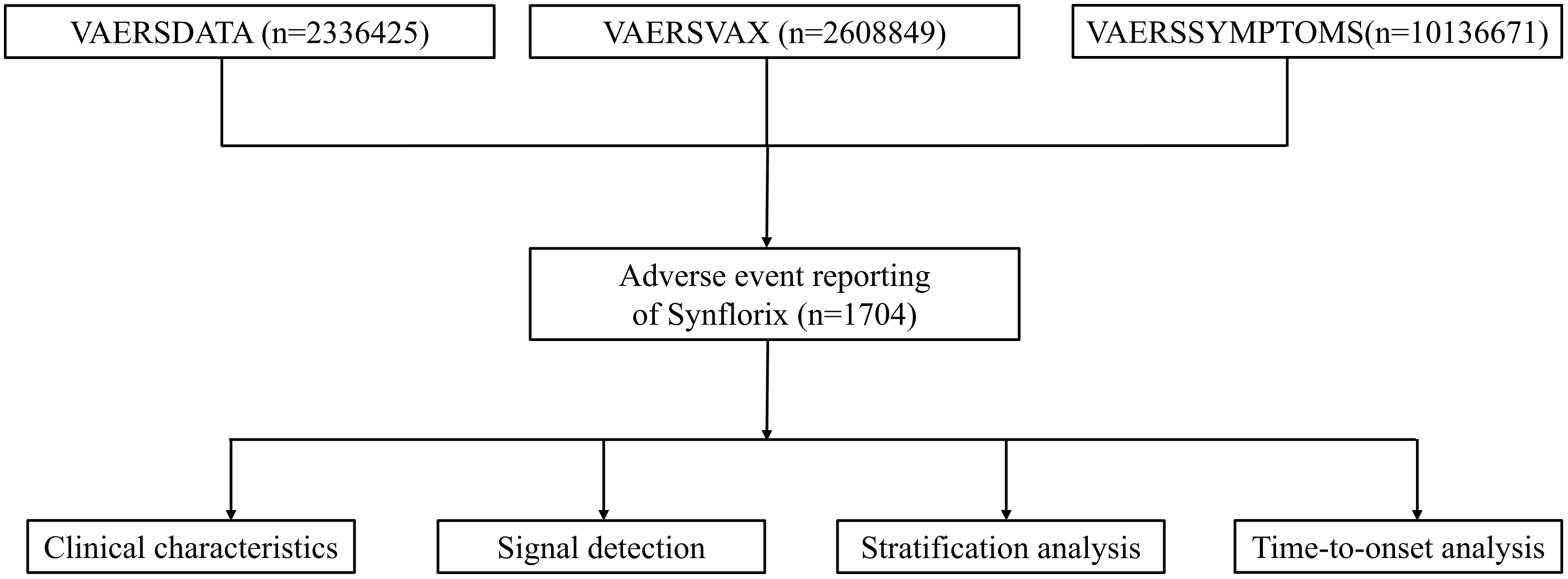
Flowchart for extracting and analyzing adverse events from VAERS database.

Synflorix received European Medicines Agency (EMA) approval in 2009 for active immunization of children aged 6 weeks to 5 years, followed by WHO prequalification in October of the same year, facilitating its introduction in multiple countries. For the present study, we extracted all VAERS reports related to Synflorix vaccination submitted between January 1, 2010, and December 30, 2024. All publicly released data have undergone de-identification and anonymization processes at the source, with legally accessible information containing no sensitive data that could identify individuals. AEs were categorized as serious or non-serious according to 21 CFR 600.80 [14]. Within the serious AE group, we further stratified events and fatality reports by age. Moreover, unstructured narrative descriptions were manually reviewed to extract specific clinical manifestations, AE symptoms, and onset times for subsequent statistical and trend analyses (Fig 1). At the same time, we compared the mortality report frequency, certain high-positive adverse reactions, and the timing of adverse events for Synflorix with data from other common pneumococcal vaccines in the VAERS database, specifically including PCV7 (PREVNAR) from 2010 to 2014, PCV13 (PREVNAR13) from 2010 to 2024, PCV15 (VAXNEUVANCE) from 2022 to 2024, PCV20 (PREVNAR20) from 2022 to 2024, and PPSV23 (PNEUMOVAX) from 2010 to 2024.

### 3. Statistical analysis

Disproportionality analysis was used as the primary data-mining approach to detect potential safety signals in VAERS. For each MedDRA PT, Reporting Odds Ratios (ROR) and 95% confidence intervals (CI) were calculated from a 2 × 2 contingency table: “A” represents the Synflorix–AE report count, while “B,” “C,” and “D” correspond to total AE counts across all vaccines (Table 1). The ROR values for PREVNAR, PREVNAR13, VAXNEUVANCE, PREVNAR20, and PNEUMOVAX were calculated in the same manner. A signal was considered positive when the lower bound of the 95% CI exceeded 1 (i.e., ROR – 1.96 × SE > 1).

**Table 1:** 2*2 table for signal detection.

	Number of target adverse reaction reports	Number of other adverse reaction reports	Total
Target drug	a	c	a + c
Other drugs	b	d	b + d
Total	a + b	c + d	a + b + c + d

a: the total number of AEs for each PT; b-d: calculate based on all the AEs across different drugs.

Initial data preparation and screening of VAERS files (VAERSDATA, VAERSSYMPTOMS, VAERSVAX) for the period from January 1, 2010, to December 30, 2024, were performed in Microsoft Excel 2021 to extract Synflorix-related records. Subsequent data manipulation and statistical analysis were carried out in R (v4.4.1) within the RStudio environment, using the dplyr package for data wrangling and the forestplot package for visualization. Forest plots were used to illustrate the ROR and its 95% CI for each common adverse reaction:


$$ROR=\frac{\left(\frac{a}{c}\right)}{\left(\frac{b}{d}\right)}=\frac{ad}{bc}$$

$$\text{9}\text{5}\%CI=e\;\text{ln}(ROR)\pm \text{1}.\text{9}\text{6}\sqrt{\left(\frac{\text{1}}{a}+\frac{\text{1}}{b}+\frac{\text{1}}{c}+\frac{\text{1}}{d}\right)}$$

$$SE(\text{ln}\;ROR)=\sqrt{\left(\frac{\text{1}}{a}+\frac{\text{1}}{b}+\frac{\text{1}}{c}+\frac{\text{1}}{d}\right)}$$

Here, “a” denotes the number of Synflorix–AE reports, “b” refers to other Synflorix reports, “c” represents reports of the same AE with other vaccines, and “d” accounts for the remaining reports. In each plot, points represent ROR estimates, horizontal bars indicate the 95% CIs, and a vertical dashed line at ROR = 1 marks the null-effect threshold. A positive signal is defined as a lower CI bound above 1.In addition, to facilitate the comparison between different pneumococcal vaccines in VAERS, we standardized certain common adverse event PTs as incidence per 100,000 reports, using the following formula [15]:


$$Frequency=\frac{number\;of\;AE\;reports}{total\;VAERS\;reports\;for\;that\;vaccine}*\text{1}\text{0}\text{0},\text{0}\text{0}\text{0}.$$

## Results

### 1. Baseline information

From January 2010 to December 2024, VAERS received 1,704 adverse event reports following Synflorix vaccination worldwide (Table 2). We excluded the 11–18 year age group from our analysis, as no vaccination records were reported for this cohort. Infants aged 0–1 year accounted for the largest share of reports, with the median age at vaccination falling within this range. The median interval from vaccination to AE onset was one day, suggesting that most reactions occurred shortly after immunization. Regarding sex distribution, 39.7% of the reports were male, 33.6% were female, and 26.7% did not specify sex. Of all the reports, 835 (49.0%) met the criteria for serious AEs.

**Table 2:** Characteristics of VAERS reports following Synflorix.

	0 ~ 1	2 ~ 10	19 ~ 54	>=55	Overall
	(N = 1054)	(N = 22)	(N = 13)	(N = 19)	(N = 1704)
**Sex**					
Female	354 (33.6%)	7 (31.8%)	7 (53.8%)	12 (63.2%)	590 (34.6%)
Male	418 (39.7%)	11 (50.0%)	6 (46.2%)	7 (36.8%)	681 (40.0%)
Unknown^a^	282 (26.7%)	4 (18.2%)	0 (0%)	0 (0%)	433 (25.4%)
**Age(years)**					
Mean (SD)	0.325 (0.293)	2.91 (1.44)	39.9 (11.5)	69.9 (8.60)	2.03 (10.1)
Median [Min, Max]	0.250 [0.0100, 1.60]	2.00 [2.00, 8.00]	42.0 [19.0, 53.0]	67.0 [55.0, 88.0]	0.250 [0.0100, 88.0]
**Numdays** ^**b**^					
Mean (SD)	5.27 (75.5)	32.5 (141)	1.17 (2.44)	3.37 (7.70)	9.67 (91.1)
Median [Min, Max]	0 [0, 2170]	0 [0, 633]	0 [0, 8.00]	1.00 [0, 33.0]	0 [0, 2170]
Missing	149 (14.1%)	2 (9.1%)	1 (7.7%)	0 (0%)	304 (17.8%)
**Serious**					
Yes	546 (51.8%)	7 (31.8%)	2 (15.4%)	2 (10.5%)	835 (49.0%)
No	508 (48.2%)	15 (68.2%)	11 (84.6%)	17 (89.5%)	869 (51.0%)

^a^gender missing(unknown); ^b^Time of adverse reactions. The overall data includes the data of age-missing groups, and thus does not equal the sum of the data of all age groups.

### 2. Detection of common adverse reaction signals

Table 3 illustrates the ten most frequently reported PTs in non-serious and serious case reports. Overall, infant crying, fever, hypotonic-hyporesponsive episodes(HHE), pallor, diarrhea, and bronchiolitis were common adverse events. Notably, “Hypotonic-hyporesponsive episode” (ROR = 83.06), “Hypotonia” (ROR = 17.33), and “Pallor” (ROR = 12.13) showed high risks of occurrence in the serious group, as well as non-serious group. Moreover, within the non-serious group, “Bronchiolitis” (ROR = 677.77) exhibited an exceptionally strong positive signal.

**Table 3:** Most Frequent Preferred Terms for serious and non-serious reports following Synflorix in VAERS with normalized frequency per 100,000 patients.

Group	PT	All	Frequency	ROR(95%Cl)
Serious	Pyrexia	369	44192	3.95 (3.55 - 4.39)
	Crying	185	22156	32.91 (28.3 - 38.27)
	Hypotonic-hyporesponsive episode	165	19760	83.06 (70.35 - 98.06)
	Vomiting	89	10659	2.28 (1.85 - 2.81)
	Hypotonia	82	9820	17.33 (13.89 - 21.63)
	Pallor	79	9461	12.13 (9.69 - 15.18)
	Diarrhoea	78	9341	3.09 (2.47 - 3.86)
	Apnoea	64	7665	35.26 (27.34 - 45.47)
	Somnolence	61	7305	7.11 (5.52 - 9.17)
	Decreased appetite	61	7305	4.19 (3.26 - 5.4)
Non-serious	Crying	342	39356	114.64 (102.26 - 128.51)
	Pyrexia	222	25547	2.27 (1.98 - 2.6)
	Hypotonic-hyporesponsive episode	137	15765	264.47 (220.88 - 316.65)
	Pallor	77	8861	11.05 (8.81 - 13.85)
	Bronchiolitis	68	7825	677.77 (515.92 - 890.41)
	Hypotonia	63	7250	53.92 (41.9 - 69.38)
	Diarrhoea	59	6789	2.74 (2.11 - 3.54)
	Vomiting	57	6559	2.04 (1.57 - 2.65)
	Restlessness	57	6559	45.35 (34.81 - 59.08)
	Somnolence	55	6329	8.31 (6.36 - 10.85)

As these are subgroup findings, the reported frequency refers to the context within this specific subgroup.

### 3. System Organ Class signal detection in serious reports

We analyzed all serious AE reports by MedDRA System Organ Class (SOC) and summarized the frequency of symptoms in Table 4. The largest category, comprising 25.93% of reports, was general disorders and administration site conditions, with pyrexia, crying, HHE, and vomiting being the most common symptoms. Other SOC categories included nervous system disorders, gastrointestinal disorders, vascular disorders, respiratory, thoracic, and mediastinal disorders, metabolism and nutrition disorders, psychiatric disorders, skin and subcutaneous tissue disorders, immune system disorders, injury, poisoning and procedural complications, infections and infestations, eye disorders, blood and lymphatic system disorders, musculoskeletal and connective tissue disorders, surgical and medical procedures, renal and urinary disorders, cardiac disorders, ear and labyrinth disorders, hepatobiliary disorders, social circumstances, congenital, familial and genetic disorders, and reproductive system and breast disorders.

**Table 4:** Distribution of adverse events in serious group by system organ class.

SOC	TOTAL	Per(%)
General disorders and administration site conditions	987	25.93
Nervous system disorders	734	19.29
Gastrointestinal disorders	320	8.41
Vascular disorders	158	4.15
Respiratory, thoracic and mediastinal disorders	251	6.59
Metabolism and nutrition disorders	112	2.94
Psychiatric disorders	228	5.99
Skin and subcutaneous tissue disorders	299	7.86
Immune system disorders	52	1.37
Injury, poisoning and procedural complications	86	2.26
Infections and infestations	229	6.02
Eye disorders	71	1.87
Blood and lymphatic system disorders	61	1.60
Musculoskeletal and connective tissue disorders	91	2.39
Surgical and medical procedures	51	1.34
Renal and urinary disorders	37	0.97
Cardiac disorders	20	0.53
Ear and labyrinth disorders	4	0.11
Hepatobiliary disorders	6	0.16
Social circumstances	2	0.05
Congenital, familial and genetic disorders	4	0.11
Reproductive system and breast disorders	3	0.08

### 4. Severely reported adverse reactions in different age groups

Recognizing that vaccine responses vary by age and immunological maturity, we stratified serious AE signals into four age cohorts (0–1, 2–10, 18–54, and ≥ 55 years) based on data-mining results (Table 5) [16]. In the 0–1 and 2–10 age groups, the ten most frequent serious AEs were similar, with injection site reactions and systemic symptoms predominating. In adults, serious AEs were primarily localized injection site reactions. Pyrexia ranked among the top ten in all age cohorts. Crying and HHE occurred exclusively in the 0–10 age groups, while chills were seen only in the 18–54 and ≥ 55 age groups.

**Table 5:** Data mining findings for reports to VAERS after vaccination with Synflorix, by age group and by serious classification.

Group	PT	All	Frequency	ROR(95%Cl)
0 ~ 1	Crying	400	37951	4.19 (3.77 - 4.65)
	Pyrexia	388	36812	1.35 (1.22 - 1.5)
	Hypotonic-hyporesponsive episode	214	20304	11.82 (10.18 - 13.71)
	Pallor	111	10531	2.59 (2.14 - 3.14)
	Hypotonia	96	9108	2.59 (2.11 - 3.18)
	Vomiting	95	9013	0.95 (0.77 - 1.16)
	Restlessness	90	8539	6.57 (5.28 - 8.18)
	Diarrhoea	79	7495	1.03 (0.82 - 1.29)
	Decreased appetite	75	7116	1.91 (1.52 - 2.41)
	Somnolence	75	7116	2.8 (2.21 - 3.53)
2 ~ 10	Crying	9	40909	4.64 (3.67)
	Pyrexia	8	36364	1.11 (0.1)
	Hypotonic-hyporesponsive episode	5	22727	6.13 (4.9)
	Gait disturbance	5	22727	3.82 (2.6)
	Pain in extremity	3	13636	2.07 (0.6)
	Injection site erythema	3	13636	−0.46 (−1.93)
	Vaccination site reaction	3	13636	6.17 (4.67)
	Vaccination site oedema	2	9091	5.75 (4.04)
	Vomiting	2	9091	0.33 (−1.37)
	Vaccination site nodule	2	9091	8.4 (6.56)
19 ~ 54	Injection site swelling	4	30769	6.89 (2.52 - 18.84)
	Erythema	4	30769	7.42 (2.71 - 20.3)
	Malaise	4	30769	6.39 (2.34 - 17.48)
	Oedema peripheral	3	23077	36.36 (11.46 - 115.33)
	Headache	3	23077	1 (0.32 - 3.19)
	Pain in extremity	3	23077	2.1 (0.66 - 6.66)
	Chills	3	23077	1.62 (0.51 - 5.12)
	Pyrexia	3	23077	1.19 (0.38 - 3.77)
	Vaccination site erythema	2	15385	25.13 (6.17 - 102.37)
	Vaccination site pain	2	15385	5.56 (1.36 - 22.62)
>=55	Erythema	7	36842	9.15 (4.21 - 19.89)
	Injection site erythema	5	26316	5.11 (2.07 - 12.65)
	Swelling	4	21053	12.7 (4.64 - 34.72)
	Pyrexia	4	21053	1.85 (0.68 - 5.06)
	Pain in extremity	4	21053	2.51 (0.92 - 6.86)
	Injection site swelling	3	15789	3.91 (1.23 - 12.41)
	Peripheral swelling	3	15789	5.94 (1.87 - 18.83)
	Dizziness	2	10526	1.86 (0.46 - 7.56)
	Pain	2	10526	1.11 (0.27 - 4.51)
	Chills	2	10526	1.17 (0.29 - 4.77)

As these are subgroup findings, the reported frequency refers to the context within this specific subgroup.

### 5. Timing of adverse reactions

**Fig 2:**
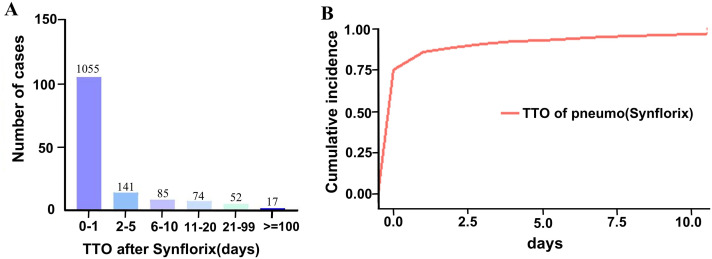
Adverse reaction of vaccination. A.Time of **occurrence of adverse reactions after** vaccination B. Cumulative **incidence of adverse reactions after vaccination.**

As shown in Fig 2A and 2B, adverse events peaked within one day of vaccination across all age groups and then declined. Table 6 presents the median onset time and interquartile range (IQR) for the ten most common AEs in the severe, non-severe, and death cohorts. In the severe group, "Pyrexia", "Crying", "Vomiting" and "Pallor" exhibited longer median onset times compared to the non-severe group: pyrexia (16.03 vs. 2.95 days); crying (6.21 vs. 0.76 days); vomiting (32.97 vs. 19.76 days); and pallor (1.58 vs. 0.39 days). Detailed onset times for other AEs are further provided in Table 6. Table 7 presents median onset times and IQRs for PT symptoms across four age groups. For instance, pyrexia’s median onset was longest in infants (0–1 year), while IQRs remained similar across age groups.

**Table 6:** Time to onset of adverse events on reports to VAERS after vaccination with Synflorix by serious classification.

Serious		Non-serious		Death	
PT	TTO(days) Median(IQR)	PT	TTO(days) Median(IQR)	PT	TTO(days) Median(IQR)
Pyrexia	16.03(0,2.00)	Pyrexia	2.95(0,1.00)	Pyrexia	1.33(0.50,2.00)
Crying	6.21(0,0)	Crying	0.76(0,0)	Crying	0(0,0)
Vomiting	32.97(0,2.00)	Vomiting	19.76(0,1.00)	Vomiting	0.50(0.25,0.75)
Somnolence	1.52(0,1.00)	Somnolence	10.77(0,0)	Somnolence	0(0,0)
Diarrhoea	8.69(0,2.00)	Diarrhoea	13.06(0,2.00)	Diarrhoea	0(0,0)
Hypotonia	0.94(0,1.00)	Hypotonia	0.85(0,0)	Autopsy	1.33(0.00,1.00)
Pallor	1.58(0,0)	Pallor	0.39(0,0)	Cardiac Arrest	0.33(0,0.50)
Hypotonic-hyporesponsive episode	0.97(0,0)	Hypotonic-hyporesponsive Episode	0.90(0,0)	Sudden Infant Death Syndrome	1.67(1.00,2.00)
Apnoea	1.03(0,1.00)	Restlessness	0.75(0,0)	Resuscitation	0(0,0)
Decreased appetite	2.08(0,1.25)	Bronchiolitis	33.03(5.00,28.00)	Respiratory Rate Increased	0(0,0)

**Table 7:** Time to onset of adverse events on reports to VAERS after vaccination with Synflorix, by age group and by serious classification.

0-1		2-10		19-54		>=55	
PT	TTO(days) Median(IQR)	PT	TTO(days) Median(IQR)	PT	TTO(days) Median(IQR)	PT	TTO(days) Median(IQR)
Pyrexia	8.51(0,1.00)	Pyrexia	0.88(0,1.00)	Pyrexia	0.50(0.25,0.75)	Pyrexia	0.50(0,1.00)
Crying	0.64(0,0)	Crying	0.22(0,0)	Erythema	0.50(0,1.00)	Erythema	7.85(1.00,9.00)
Hypotonic-hyporesponsive episode	0.45(0,0)	Hypotonic-hyporesponsive episode	0.60(0,1.00)	Malaise	0(0,0)	Swelling	9.00(0.75,9.75)
Pallor	0.96(0,0)	Gait disturbance	0(0,0)	Chills	0(0,0)	Chills	0(0,0)
Hypotonia	0.58(0,0)	Pain in extremity	0(0,0)	Pain in extremity	0.67(0.50,1.00)	Pain in extremity	0.25(0,0.25)
Restlessness	0.60(0,0)	Injection site erythema	0(0,0)	Injection site swelling	0(0,0)	Injection site swelling	0.67(0,1.00)
Vomiting	31.64(0,0)	Vomiting	0.50(0.25,0.75)	Oedema peripheral	0.33(0,0.50)	Peripheral swelling	0.67(050,1.00)
Diarrhoea	1.03(0,1.00)	Vaccination site oedema	0(0,0)	Headache	0.33(0,0.50)	Dizziness	1.50(1.25,1.75)
Decreased appetite	1.00(0,1.00)	Vaccination site reaction	0.33(0,0.50)	Vaccination site erythema	4.00(4.00,4.00)	Pain	4.50(2.75,6.25)
Somnolence	0.93(0,0)	Vaccination site nodule	316.50 (158.25,474.75)	Vaccination site pain	4.00(4.00,4.00)	Injection site erythema	0.40(0,0)

### 6. Detection of adverse reaction signals in fatal cases from serious reports

**Fig 3:**
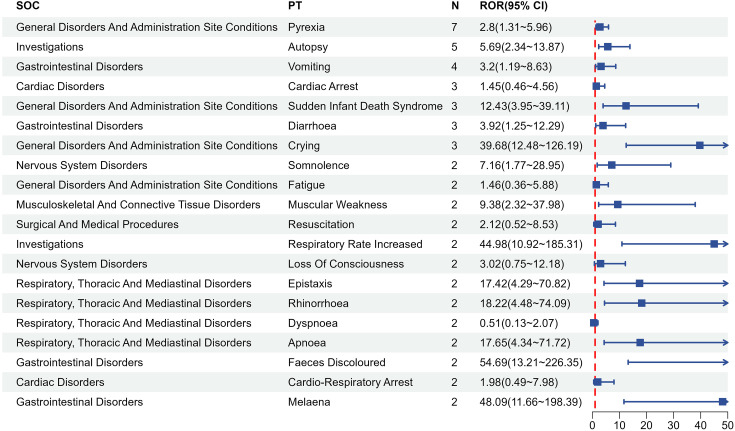
Distribution of adverse events by system organ class among 33 death reports after Synflorix in VAERS.

VAERS documented 33 fatal reports associated with Synflorix vaccination, five of which included autopsy findings, while the remaining cases were supported by death certificates and/or medical records. Pyrexia was the most commonly reported AE. Fig 3 lists the top 20 PTs identified in fatal cases, which include febrile events, gastrointestinal disorders, infections and infestations, and nervous system disorders occurring shortly after vaccination. Gastrointestinal AEs were particularly prominent, with vomiting (ROR = 3.20), diarrhea (ROR = 3.92), and melaena (ROR = 48.09) showing the strongest disproportionality signals.

### 7. Comparison of reported fatalities, number of specific PT reports, and ROR values between Synflorix and other pneumococcal vaccines in VAERS

Table 8 shows that Synflorix has the third-highest frequency of mortality reports (1,937), following PREVNAR with 3,161 reports and Prevnar 13 with 2,500 reports per 100,000 VAERS reports. In addition, Synflorix also has relative higher frequencies for several adverse events compared to other vaccines. Specifically, Synflorix has the highest frequencies for HHE (17723), hypotonia (8509), pallor (9155), and bronchiolitis (4167), compared to other pneumococcal vaccines. Additionally, Synflorix’s ROR and 95% CI are also relatively higher than the other four pneumococcal vaccines. These results indicate that Synflorix has a somewhat higher mortality report frequency, and its PTs (HHE, hypotonia, pallor, bronchiolitis) have relative higher frequencies and ROR than the other pneumococcal vaccines in the VAERS database.

**Table 8:** Comparison of number of mortality reports, specific PT reports and ROR values between Synflorix and other pneumococcal vaccines in VAERS.

Vaccine	Total number of reports	Number of reported deaths (Frequency)	HHE		Hypotonia		Pallor		Bronchiolitis	
			Case (Frequency)	ROR(95%Cl)	Case (Frequency)	ROR(95%Cl)	Case (Frequency)	ROR(95%Cl)	Case (Frequency)	ROR(95%Cl)
PCV7(PREVNAR)	6074	192 (3161)	86 (1416)	*13.77 (11.1 - 17.09)	280 (4610)	*21.36 (18.92 −24.1)	344 (5663)	*6.84 (6.14 - 7.61)	17 (280)	*12.95 (7.98- 21)
PCV10 (Synflorix)	1704	33 (1937)	302 (17723)	*163.08 (144.36 - 184.22)	145 (8509)	*33.84 (28.66 - 39.97)	156 (9155)	*9.78 (8.34 - 11.46)	71 (4167)	*191.44 (148.91- 246.13)
PCV13 (PREVNAR13)	43273	1082 (2500)	431 (996)	*11.34 (10.22 - 12.59)	1113 (2572)	*14.25 (13.33 - 15.22)	1338 (3092)	*3.91 (3.7 - 4.14)	77 (178)	*9.37 (7.35- 11.94)
PCV15 (VAXNEUVANCE)	1034	19 (1838)	6 (580)	*7.49 (3.36 - 16.71)	16 (1547)	*9.3 (5.69 - 15.22)	19 (1838)	*2.98 (1.9 - 4.67)	1 (97)	1.1 (0.15 - 7.79)
PCV20 (PREVNAR20)	4350	43 (989)	2 (46)	*0.49 (0.12 - 1.97)	14 (322)	1.6 (0.95 - 2.71)	39 (897)	1.21 (0.88 - 1.65)	2 (46)	0.43 (0.11 - 1.74)
PPSV23 (PNEUMOVAX, PNU-IMUNE)	49647	254 (512)	5 (10)	0.11 (0.04 - 0.26)	27 (54)	0.27 (0.18 - 0.39)	126 (254)	0.34 (0.28 - 0.4)	28 (56)	*3.02 (2.06 - 4.42)

Asterisks (*) indicate statistically significant signals in algorithm;

### 8. Comparison of adverse reaction incidence and cumulative incidence after vaccination for Synflorix and other pneumococcal vaccines

**Fig 4:**
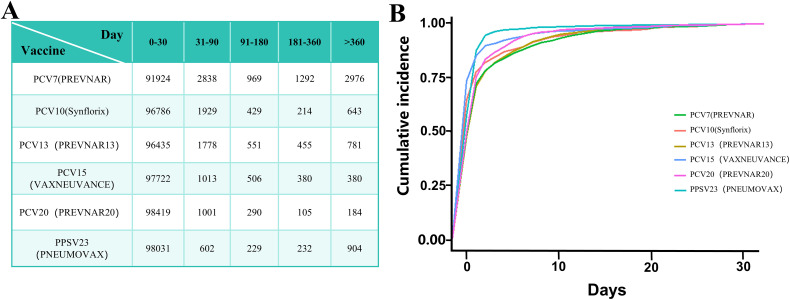
Comparison of adverse reaction incidence and cumulative incidence after vaccination for Synflorix and Other pneumococcal vaccines. A.Time to onset of adverse reactions after vaccination (frequency, per 100,000 individuals) B. Cumulative incidence of adverse reactions after vaccination.

We compared the incidence and cumulative incidence of adverse reactions for Synflorix with several other commonly used pneumococcal vaccines, including Prevnar, Prevnar 13, Vaxneuvance, Prevnar 20, and Pneumovax. Fig 4A shows the standardized incidence rates (per 100,000 reports) for each vaccine across different time intervals (0–30 days, 31–90 days, 91–180 days, 181–360 days, and >360 days). It indicates that Synflorix had incidence rates in the early post-vaccination period (0–30 days) that were similar to those of the other vaccines. This suggests that the frequency of adverse events shortly after vaccination was comparable across these vaccines. Fig 4B shows the cumulative incidence of adverse reactions after vaccination, with Synflorix displaying a similar cumulative incidence to the other vaccines, showing no significant differences.

## Discussion

Vaccination is essential for preventing infectious diseases and protecting public health. With growing attention to vaccine safety, we analyzed AEs following Synflorix vaccination using VAERS data from 2010 to 2024. Our baseline analysis shows that the highest number of adverse events occurred in the 0–1 year age group, with fewer events reported in other age groups. The observed data coincides with the majority of vaccine recipients being infants, alongside the possibility of dosing issues. Among the top ten PTs reported in both non-serious and serious AEs, most were consistent with those listed in the Synflorix package insert: pyrexia, crying, vomiting, diarrhea, apnea, lethargy, decreased appetite, and restlessness. Nonetheless, we also identified PTs not included in the insert: HHE, hypotonia, and pallor in severe cases, and bronchiolitis in non-serious cases.

HHE is characterized by a sudden loss of muscle tone, reduced consciousness, and pallor or cyanosis within 48 hours post-vaccination, primarily in children under 10 years of age [17,18]. It typically resolves without intervention and has no lasting effects [19,20]. While its mechanism remains elusive, it may involve pediatric immune responses and components of the vaccine [21]. HHE has been reported after diphtheria-tetanus-pertussis (DTP), Haemophilus influenzae type b (Hib), and hepatitis B vaccines [22,23]. A Chinese study also observed HHE following the 13-valent pneumococcal conjugate vaccine, which aligns with our findings [24].

Bronchiolitis is a lower respiratory tract infection primarily affecting infants and young children, often linked to Respiratory Syncytial Virus (RSV) infection [25,26]. Its clinical symptoms include cough, wheezing, shortness of breath, and feeding difficulties, which can lead to respiratory distress or hypoxemia in severe cases [27,28]. This study reports the occurrence of bronchiolitis as a common adverse reaction following Synflorix vaccination, with an ROR value of 191.44. The standardized report count and ROR value for bronchiolitis were relative higher than those for other pneumococcal vaccines in the VAERS database. Based on these findings, it is recommended that healthcare providers remain vigilant for symptoms related to bronchiolitis during Synflorix vaccination and advise timely medical attention if such symptoms occur [29].

When examining onset timing, pyrexia, crying, vomiting, somnolence, and diarrhea were observed across the serious, non-serious, and fatal groups. The fatal group had the shortest median onset times, emphasizing the need for close monitoring during the first 24 hours post-vaccination and prompt medical evaluation if these AEs occur. In contrast, non-fatal groups experienced delayed cases of bronchiolitis and vomiting, sometimes weeks after immunization, suggesting that observation should extend up to 30 days. Age-stratified analysis revealed that children experienced more frequent and systemic severe adverse events than adults, likely due to their developing immune and neurological systems, as well as their limited ability to report symptoms. In infants (0–1 year), reactions such as crying, HHE, pallor, and vomiting occurred acutely, typically within one day post-vaccination, with fever occasionally having a delayed onset. In children aged 2–10 years, limb pain, motor disturbances, transient fever, and occasional injection-site nodules were reported over a longer timeframe. For adults (≥18 years), adverse events were predominantly localized to the injection site within ten days, with rare systemic symptoms. Data for individuals aged 55 and older remain limited, so these observations should be considered preliminary and interpreted with caution [2].

In the comparative analysis with other pneumococcal vaccines, Synflorix showed the third-highest frequency of mortality and certain specific PTs (HHE, hypotonia, pallor, Bronchiolitis) compared to other pneumococcal vaccines. The primary difference among these pneumococcal vaccines lies in their serotype coverage. Synflorix covers 10 serotypes (including 1, 3, 4, 6B, 9V, 14, 18C, 19F, 23F, and 7F). In contrast, Prevnar 13 (PCV13) adds 6A, 19A, and 18B; Vaxneuvance (PCV15) adds 22F and 33F to PCV13; Prevnar 20 (PCV20) further adds 8, 10A, 12F, 15B, 20, 22F, and 33F; and Pneumovax 23 (PPSV23) covers the most, with 23 serotypes, including all of the aforementioned serotypes and additional serotypes 2, 5, 7F, 11A, 12F, 15B, and 17F [30]. Although high-valent vaccines provide more comprehensive immune coverage, studies suggest that the immune response from these vaccines is more dispersed, with weaker IgG antibody responses, potentially leading to a lower frequency of specific adverse reactions [1,31]. In the comparison of adverse reaction onset times, Synflorix shared a similar pattern with all other pneumococcal vaccines, with reactions predominantly concentrated in the short-term post-vaccination period.

This VAERS-based study systematically described the distributions of adverse event timing and symptom profiles across different age groups, addressing a gap in multi-age safety data for Synflorix. Furthermore, in comparisons with other common pneumococcal vaccines within the VAERS database, we observed higher reporting frequencies and RORs for HHE, hypotonia, pallor, and bronchiolitis following Synflorix vaccination. These reactions warrant increased attention in future Synflorix immunization practices. Regarding limitations, VAERS is a passive surveillance system and is therefore subject to underreporting, selective reporting, and selection bias. Key clinical details are often missing, and the quality of reports varies considerably. Since the database lacks a verifiable population denominator and an unvaccinated comparator group, the true incidence of events cannot be estimated, and causal inference cannot be established. Future research should involve well-controlled epidemiological studies to further investigate and confirm any potential associations.

## Conclusion

Our analysis of Synflorix-related VAERS reports from January 2010 to December 2024 identified new adverse reaction signals, including HHE, hypotonia, pallor, and bronchiolitis. The notably shorter median onset time in fatal cases emphasizes the need for increased vigilance within the first 24 hours after vaccination. Comparisons between severe and non-severe events revealed that severe reactions occur later. Age-stratified trends showed distinct temporal and clinical patterns across different age groups, highlighting the importance of targeted monitoring strategies. When compared to other pneumococcal vaccines within the VAERS database, Synflorix showed the third-highest frequency of mortality frequency andrelatively higher RORs for HHE, hypotonia, pallor, and bronchiolitis. These findings support enhanced post-vaccination monitoring, particularly for younger populations and those with underlying conditions.

## Supporting information

S1 File Raw data (ID PONE-D-25–29249). The original code can be downloaded via https://github.com/llyy-cloud/The-code-of-PONE-D-25-29249.git.
(ZIP)

## References

[1] Feng S, McLellan J, Pidduck N, Roberts N, Higgins JP, Choi Y, et al. Immunogenicity and seroefficacy of pneumococcal conjugate vaccines: a systematic review and network meta-analysis. Health Technol Assess. 2024;28(34):1–109. doi: 10.3310/YWHA3079 39046101 PMC11284620

[2] Garcia Quesada M, Peterson ME, Bennett JC, Hayford K, Zeger SL, Yang Y, et al. Serotype distribution of remaining invasive pneumococcal disease after extensive use of ten-valent and 13-valent pneumococcal conjugate vaccines (the PSERENADE project): a global surveillance analysis. Lancet Infect Dis. 2025;25(4):445–56. doi: 10.1016/S1473-3099(24)00588-7 39706205 PMC11947070

[3] Plosker GL. 10-Valent pneumococcal non-typeable haemophilus influenzae protein D-conjugate vaccine: a review in infants and children. Paediatr Drugs. 2014;16(5):425–44. doi: 10.1007/s40272-014-0089-x 25192686

[4] Croxtall JD, Keating GM. Pneumococcal polysaccharide protein D-conjugate vaccine (Synflorix; PHiD-CV). Paediatr Drugs. 2009;11(5):349–57. doi: 10.2165/11202760-000000000-00000 19725600

[5] Leach AJ, Wilson N, Arrowsmith B, Beissbarth J, Mulholland EK, Santosham M, et al. Otitis media at 6-monthly assessments of Australian First Nations children between ages 12-36 months: Findings from two randomised controlled trials of combined pneumococcal conjugate vaccines. Int J Pediatr Otorhinolaryngol. 2023;175:111776. doi: 10.1016/j.ijporl.2023.111776 37951020

[6] Domingues CMAS, Verani JR, Montenegro Renoiner EI, de Cunto Brandileone MC, Flannery B, de Oliveira LH, et al. Effectiveness of ten-valent pneumococcal conjugate vaccine against invasive pneumococcal disease in Brazil: a matched case-control study. Lancet Respir Med. 2014;2(6):464–71. doi: 10.1016/S2213-2600(14)70060-8 24726406 PMC9003592

[7] Varghese L, Talbot L, Govender A, Zhang X-H, Mungall BA. A cost-effectiveness analysis of the 10-valent pneumococcal non-typeable haemophilus influenzae protein D CONJUGATE VACCINE (PHiD-CV) compared to the 13-valent pneumococcal conjugate vaccine (PCV13) for universal mass vaccination implementation in New Zealand. Appl Health Econ Health Policy. 2018;16(3):331–45. doi: 10.1007/s40258-018-0387-5 29633160 PMC5940727

[8] Marijam A, Schuerman L, Izurieta P, Pereira P, Van Oorschot D, Mehta S, et al. Estimated public health impact of human rotavirus vaccine (HRV) and pneumococcal polysaccharide protein D-conjugate vaccine (PHiD-CV) on child morbidity and mortality in Gavi-supported countries. Hum Vaccin Immunother. 2022;18(7):2135916. doi: 10.1080/21645515.2022.2135916 36507685 PMC9766466

[9] Castiglia P, Pradelli L, Castagna S, Freguglia V, Palù G, Esposito S. Overall effectiveness of pneumococcal conjugate vaccines: An economic analysis of PHiD-CV and PCV-13 in the immunization of infants in Italy. Hum Vaccin Immunother. 2017;13(10):2307–15. doi: 10.1080/21645515.2017.1343773 28700264 PMC5647981

[10] Farrar JL, Childs L, Ouattara M, Akhter F, Britton A, Pilishvili T, et al. Systematic review and meta-analysis of the efficacy and effectiveness of pneumococcal vaccines in adults. Pathogens. 2023;12(5):732. doi: 10.3390/pathogens12050732 37242402 PMC10222197

[11] Zhou H, Yang J, Zhang J, Liu P, Yao D. A real-world pharmacovigilance analysis of hepatitis B vaccine using the U.S. Vaccine Adverse Event Reporting System (VAERS) database. Sci Rep. 2025;15(1):6022. doi: 10.1038/s41598-025-90135-8 39972053 PMC11840001

[12] Gordon ER, Adeuyan O, Kwinta BD, Schreidah CM, Fahmy LM, Queen D, et al. Exploring cutaneous lymphoproliferative disorders in the wake of COVID-19 vaccination. Skin Health Dis. 2024;4(3):e367. doi: 10.1002/ski2.367 38846690 PMC11150739

[13] Alderson MR. Status of research and development of pediatric vaccines for Streptococcus pneumoniae. Vaccine. 2016;34(26):2959–61. doi: 10.1016/j.vaccine.2016.03.107 27083428 PMC4906266

[14] Myers TR, McNeil MM, Ng CS, Li R, Marquez PL, Moro PL, et al. Adverse events following quadrivalent meningococcal diphtheria toxoid conjugate vaccine (Menactra®) reported to the Vaccine Adverse Event Reporting System (VAERS), 2005-2016. Vaccine. 2020;38(40):6291–8. doi: 10.1016/j.vaccine.2020.07.039 32747215 PMC7495357

[15] Ricke DO, Smith N. VAERS vasculitis adverse events retrospective study: etiology model of immune complexes activating fc receptors in kawasaki disease and multisystem inflammatory syndromes. Life (Basel). 2024;14(3):353. doi: 10.3390/life14030353 38541678 PMC10971466

[16] Kim Y, Han K, Kim JH. Retinal Vascular Occlusions After COVID-19 Vaccination in South Korea: A Nation-Wide Population-Based Study. Ophthalmic Epidemiol. 2025;32(4):403–11. doi: 10.1080/09286586.2024.2399345 39288331

[17] DuVernoy TS, Braun MM, the VAERS Working Group. Hypotonic-hyporesponsive episodes reported to the Vaccine Adverse Event Reporting System (VAERS), 1996-1998. Pediatrics 2000;106:e52–e52. doi: 10.1542/peds.106.4.e52 11015547

[18] Puente Gómez I, Verheust C, Hanssens L, Dolhain J. Safety profile of Infanrix hexa - 17 years of GSK’s passive post-marketing surveillance. Expert Rev Vaccines. 2020;19(8):771–9. doi: 10.1080/14760584.2020.1800458 32729745

[19] Buettcher M, Heininger U, Braun M, Bonhoeffer J, Halperin S, Heijbel H, et al. Hypotonic-hyporesponsive episode (HHE) as an adverse event following immunization in early childhood: case definition and guidelines for data collection, analysis, and presentation. Vaccine. 2007;25(31):5875–81. doi: 10.1016/j.vaccine.2007.04.061 17537554

[20] Hansen J, Decker MD, Lewis E, Fireman B, Pool V, Greenberg DP, et al. Hypotonic-hyporesponsive Episodes After Diphtheria, Tetanus and Acellular Pertussis Vaccination. Pediatr Infect Dis J. 2021;40(12):1122–6. doi: 10.1097/INF.0000000000003308 34420008 PMC8575166

[21] Velasco J, Montero DA, Guzmán M. Hypotonic-Hyporesponsive Episode after immunization with whole-cell pertussis combination vaccine. Clinical Case Report. Rev Chil Pediatr. 2017;88(6):771–5. doi: 10.4067/S0370-41062017000600771 29546927

[22] Vermeer-de Bondt PE, van der Maas NAT. The effect of age and dose number on the risk of collapse (hypotonic-hyporesponsive episode) after pertussis vaccination. Pediatr Infect Dis J. 2008;27(4):355–7. doi: 10.1097/INF.0b013e318162a127 18379375

[23] Martins RM, Camacho LAB, Lemos MCF, Noronha TG de, Carvalho MHC de, Greffe N, et al. Incidence of hypotonic-hyporesponsive episodes associated to the combined DTP/Hib vaccine used in Brazilian National Immunizations Program. J Pediatr (Rio J). 2007;83(6):523–8. doi: 10.2223/JPED.1721 18074056

[24] Hu Y, Pan X, Chen F, Wang Y, Liang H, Shen L, et al. Surveillance of adverse events following immunization of 13-valent pneumococcal conjugate vaccine among infants, in Zhejiang province, China. Hum Vaccin Immunother. 2022;18(1):2035141. doi: 10.1080/21645515.2022.2035141 35240930 PMC9009923

[25] Vila J, Lera E, Peremiquel-Trillas P, Andrés C, Martínez L, Barceló I, et al. Increased RSV-A bronchiolitis severity in RSV-infected children admitted to a reference center in Catalonia (Spain) between 2014 and 2018. J Pediatric Infect Dis Soc. 2023;12(3):180–3. doi: 10.1093/jpids/piad009 36744919

[26] Adar A, Goldbart AD, Burrack N, Geva N, Cohen B, Golan-Tripto I. C-reactive protein is associated with severity in hospitalized children with respiratory syncytial virus bronchiolitis. Isr Med Assoc J. 2025;27(3):165–71. 40134169

[27] Flaherty BF, Olsen CS, Coon ER, Srivastava R, Cook LJ, Keenan HT. Patterns of use of β-2 agonists, steroids, and mucoactive medications to treat bronchiolitis in the PICU: U.S. Pediatric Health Information System 2009-2022 Database Study. Pediatr Crit Care Med. 2025;26(3):e294–303. doi: 10.1097/PCC.0000000000003670 40048297 PMC11889393

[28] Chen Y, Liang S, Lu Y, Zhou X, Zheng R, Chen Y. Case Report: First report of Legionella micdadei pneumonia and organizing pneumonia in a patient with myelodysplastic and Sweet syndromes. Front Immunol. 2025;16:1510948. doi: 10.3389/fimmu.2025.1510948 40051621 PMC11882538

[29] Kikuchi R, Iwai Y, Watanabe Y, Nakamura H, Aoshiba K. Acute respiratory failure due to eosinophilic pneumonia following pneumococcal vaccination. Hum Vaccin Immunother. 2019;15(12):2914–6. doi: 10.1080/21645515.2019.1631134 31184980 PMC6930081

[30] Schellenberg JJ, Adam HJ, Baxter MR, Karlowsky JA, Golden AR, Martin I, et al. Comparison of PCV10, PCV13, PCV15, PCV20 and PPSV23 vaccine coverage of invasive Streptococcus pneumoniae isolate serotypes in Canada: the SAVE study, 2011-20. J Antimicrob Chemother. 2023;78(Suppl 1):i37–47. doi: 10.1093/jac/dkad068 37130588

[31] Martinón-Torres F, Martinez SN, Kline MJ, Drozd J, Trammel J, Peng Y, et al. A phase 3 study of 20-valent pneumococcal conjugate vaccine in healthy toddlers previously vaccinated in infancy with 13-valent pneumococcal conjugate vaccine. Vaccine. 2025;53:126931. doi: 10.1016/j.vaccine.2025.126931 40081152

